# [Retracted] lncRNA‑CD160 decreases the immunity of CD8^+^ T cells through epigenetic mechanisms in hepatitis B virus infection

**DOI:** 10.3892/ol.2025.15075

**Published:** 2025-05-06

**Authors:** Jiansong Wu, Qiang Niu, Jie Yuan, Xiaodan Xu, Liuxia Cao

Oncol Lett 20: 235–247, 2020; DOI: 10.3892/ol.2020.11534

Following the publication of this paper, it was drawn to the Editor's attention by a concerned reader that the FISH and immunofluorescence assay data shown in Fig. 4J and L, and certain of the immunohistochemical data in Fig. 5D, were strikingly similar to data that had already appeared in previously published articles written by different authors at different research institutes. Owing to the fact that the contentious data in the above article had already been published prior to its submission to *Oncology Letters*, the Editor has decided that this paper should be retracted from the Journal. The authors were asked for an explanation to account for these concerns, but the Editorial Office did not receive a reply. The Editor apologizes to the readership for any inconvenience caused.

